# Food reward in the obese and after weight loss induced by calorie restriction and bariatric surgery

**DOI:** 10.1111/j.1749-6632.2012.06573.x

**Published:** 2012-05-22

**Authors:** Hans-Rudolf Berthoud, Huiyuan Zheng, Andrew C Shin

**Affiliations:** Neurobiology of Nutrition Laboratory, Pennington Biomedical Research Center, Louisiana State University SystemBaton Rouge, Louisiana

**Keywords:** obesity, diabetes, palatable food, hedonic eating, food addiction, liking, wanting, motivation, mesolimbic dopamine system

## Abstract

Increased availability of tasty, energy-dense foods has been blamed as a major factor in the alarmingly high prevalence of obesity, diabetes, and metabolic disease, even in young age. A heated debate has started as to whether some of these foods should be considered addictive, similar to drugs and alcohol. One of the main arguments for food addiction is the similarity of the neural mechanisms underlying reward generation by foods and drugs. Here, we will discuss how food intake can generate reward and how behavioral and neural reward functions are different in obese subjects. Because most studies simply compare lean and obese subjects, it is not clear whether predisposing differences in reward functions cause overeating and weight gain, or whether repeated exposure or secondary effects of the obese state alter reward functions. While studies in both rodents and humans demonstrate preexisting differences in reward functions in the obese, studies in rodent models using calorie restriction and gastric bypass surgery show that some differences are reversible by weight loss and are therefore secondary to the obese state.

## Introduction

Obesity and its comorbidities have been recognized as a major global health problem. Pharmacological treatment has come to an impasse, with only moderately effective drugs available now and few promising ones on the horizon. This has prompted reconsideration of other treatment avenues, including behavioral therapies, bariatric surgery, and various electrical stimulation devices. Dieting, although effective in producing weight loss and health improvements, has been proven very difficult to adhere to over time because calorie restriction–induced biological adaptations evoke strong feelings of hunger and craving for food, eventually overpowering restraint (e.g., Ref. 1). These strong adaptive responses do not seem to kick in after weight loss induced by bariatric surgeries, particularly Roux-en-Y gastric bypass (RYGB) surgery, so that obesity surgery has rapidly advanced to the most effective available long-term treatment for morbidly obese and moderately obese patients with type 2 diabetes (e.g., Ref. 2).

Ideal treatments attack the cause, rather than the symptoms, of a disease. Clearly, the cause of obesity in a large majority of patients cannot easily be identified because it is multifactorial. Although it is widely accepted that an environment of readily accessible processed foods and a sedentary lifestyle are major causative factors, it is the interaction of these factors with genetic predisposition that is important for the expression of obesity.^3^ These predisposing traits are likely due to a great number of gene variants, as well as epigenetic and other early life-programming processes.^4^ Ultimately, only systematic analyses with controlled environmental conditions in genetically identified individuals using prospective, rather than cross-sectional, approaches will be able to dissect the true causes of obesity; further, such requirements are much easier met in animals than in human subjects.

One of the key ingredients for the development of obesity in the modern world appears to be overindulgence in foods that are palatable, easily available, rich in calories from fat and sugar, and often poor in micronutrients. Because such foods are rewarding by generating pleasure and satisfaction, particularly in a world that for many is becoming increasingly stressful, the term “food addiction” has been used in analogy to drug addiction.^5^,^6^ It has been suggested that obesity can result from an attraction or addiction to, and overconsuming of, such high calorie junk food. However, cause and effect in the relationship between the availability and overconsumption of such foods and the development of obesity are far from clear. Is the increased availability and/or the repeated exposure to highly rewarding foods more important than differences in the brain reward system? Does the obese state with its wide-ranging alterations in hormonal and inflammatory signaling secondarily affect the food reward system? These are questions that ultimately have to be answered in order to reach a clearer understanding of cause and effect and that will allow the design of better behavioral, surgical, and pharmacological treatments for obesity.

In the following review, we will first briefly discuss the basic concepts and the neural underpinnings of food reward, as well as the limited human data on the relationship between obesity and food reward processes. As an initial attempt to dissociate cause and effect, we will then summarize and discuss some of our own published data on this relationship in several rat models of obesity and weight loss.

## Definition of food reward

It is thought that instinctual behaviors that are essential for survival have evolved over millions of years, and that their neural control mechanisms are particularly powerful.^7^,^8^ Especially in warm-blooded animals, finding and eating food is a daily necessity that is very high in the hierarchy of instinctive behaviors even when scarce and dangerous environmental conditions must be overcome. Food reward has been suggested to provide the necessary motivation to overcome such conditions. Thus, food is a powerful natural reinforcer that out-competes most other behaviors, particularly when metabolically hungry. Ingestive behavior consists of procurement, consummatory, and postconsummatory phases,^9^ and each of these three phases contributes to reward, which then can guide future behaviors.

In the procurement phase, the decision-making process responsible for switching attention is central to the modern field of neuroeconomics, and reward expectancy is perhaps the main factor determining the outcome of this process–response selection. To make this choice, the brain uses representations of reward expectancy and effort/risk-requirement from prior experiences to optimize cost benefit.^10^–^14^

During the consummatory phase, immediate sensory attributes of the goal object such as seeing, smelling, and ultimately tasting the first bite of the food provide the first feedback to its predicted reward value and may acutely enhance its motivating power. Appetite is typically augmented by the generation of cephalic phase responses such as gastric acid and insulin secretion.^15^ During eating, immediate, direct pleasure is derived from mainly gustatory and olfactory sensations, driving consumption throughout the meal until satiation signals dominate.^16^ The length of the consummatory phase is highly variable, as it takes only a few minutes to devour a hamburger, but it may take hours to savor a five-course meal. During such longer meals, ingested food increasingly engages postoral reward processes that interact with oral reward.

The postingestive phase is probably the most complex and least understood phase of ingestive behavior in terms of reward processing. Nutrient sensors in the gastrointestinal tract and elsewhere in the body also contribute to the generation of food reward during and after a meal.^17^,^18^ The same taste receptors found in the oral cavity are also expressed in gut epithelial cells 19 and in the hypothalamus.^20^ But even when all taste processing is eliminated by genetic manipulation, mice still learn to prefer sugar over water, suggesting the generation of food reward by processes of glucose utilization.^18^ Rather than the acute pleasure of tasty food in the mouth, there is a general feeling of satisfaction that lingers on long after termination and most likely contributes to the reinforcing power of a meal. Thus, a variety of sensory stimuli and emotional states or feelings with vastly different temporal profiles make up the rewarding experience of eating, and the underlying neural functions are only beginning to be understood.

## Components of reward functions and their neural mechanisms

The neural mechanisms and behavioral manifestations of reward functions as they pertain to natural food reward and its similarities to drug reward have been subject to excellent reviews^5^–^7^,^21^ and are discussed only briefly here. Berridge and Robinson have parsed reward into separable psychological and neural components, liking, wanting, and learning.^22^ The characteristic orofacial expressions displayed by decerebrate rats^23^ and anencephalic infants^24^ in response to sweet taste strongly suggest that the forebrain is not the only brain area involved in experiencing the hedonic impact or liking of pleasant stimuli. Berridge and Robinson^22^ refer to these expressions as objective affective reactions or implicit affect and to the psychological process as implicit *liking*. Besides neural circuits in the hindbrain, the nucleus accumbens and ventral pallidum in the limbic forebrain appear to be some of the other key components of the distributed neural network mediating liking of palatable foods. The mu-opioid receptor appears to play a crucial role. Local injection of the selective mu-opioid agonist DAMGO into the nucleus accumbens elicits voracious food intake, particularly of palatable sweet or high-fat foods.^25^–^27^ This increased consumption of highly palatable foods appears to be due to increased liking, as morphine microinjections into this area increased the number of positive affective reactions,^28^ and microinjection of a selective mu-opioid antagonist reduced sucrose drinking.^29^ The most sensitive area for this effect was the caudal shell of the nucleus accumbens, near the border with the adjacent core.^28^ We have recently demonstrated that nucleus accumbens injection of a mu-opioid receptor antagonist transiently suppressed such sucrose-evoked positive hedonic orofacial reactions.^30^

In humans, subjective liking can be assessed by questionnaires and visual analog scales. In the Power of Food Scale (PFS), appetite for palatable food items is estimated by asking subjects how much they would like to eat certain foods when they were available, when they are present in front of their eyes, and when they are actually tasted, but not ingested.^31^ These three levels of proximity clearly generate different neural response patterns, involving more or less visual, taste, and olfactory processing. To consciously experience and give subjective ratings of pleasure from palatable foods (liking), humans very likely use areas in the prefrontal and cingulate cortex.^32^ Thus, the neurological substrate responsible for liking palatable food items is distributed throughout the neuraxis and cannot be conveniently eliminated by lesions. One of the common denominators of the distributed network may be opioidergic transmission, particularly through the mu-opioid receptor.

Another component of reward is motivation or *wanting*. Typically, motivation comes to fruition by “going for” something that has generated pleasure in the past through a learning process—wanting what we like. However, wanting can also be dissociated from liking as demonstrated by sodium-depleted rats wanting hypertonic saline, a taste they had never “liked” before and also by drug addicts that no longer like to inject themselves.^33^,^34^ Dopamine signaling within the mesolimbic dopamine projection system appears to be a crucial component of this process. Phasic activity of dopamine neuron projections from the ventral tegmental area to the nucleus accumbens in the ventral striatum are specifically involved in the decision-making process during the preparatory (appetitive) phase of ingestive behavior.^10^,^35^ In addition, when palatable foods such as sucrose are actually consumed, a sustained and sweetness-dependent increase occurs in nucleus accumbens dopamine levels and turnover.^36^–^38^ Dopamine signaling in the nucleus accumbens thus appears to play a role in both the preparatory and consummatory phases of an ingestive bout. The nucleus accumbens shell is thereby part of a neural loop including the lateral hypothalamus and the ventral tegmental area, with orexin neurons playing a key role.^8^,^39^–^46^ This loop is likely important for the attribution of incentive salience to goal objects by metabolic state and other need signals available to the lateral hypothalamus, as discussed below.

In summary, although there have been excellent recent attempts to separate its components, the functional concept and neural circuitry underlying food reward is still poorly defined. Specifically, it is not well understood how reward, generated during anticipation, consummation, and satiation, are computed and integrated. Future research with modern neuroimaging techniques in humans and invasive neurochemical analyses in animals will be necessary for a more complete understanding.

## Reward functions in the obese

### Liking

A popular assumption is that obese individuals like food more than lean individuals and that this increased liking results in overeating and eventually obesity. Obese subjects report higher hedonic hunger, as measured with the PFS^47^–^49^ and higher liking for a given sweetness^50^ compared with normal weight subjects. Interestingly, this is in spite of decreased perceived sweetness in obese subjects.^50^ Thus, as concluded by Bartoshuk *et al*.,^50^ liking increases as a function of sweetness more in obese subjects and more as BMI increases, and for the same perceived sweetness, liking increases as BMI increases. Importantly, in underweight subjects with a BMI of <18.5, liking did not increase as a function of perceived sweetness.^50^ Furthermore, subjects with a tendency for binge eating showed increased liking for all food categories.^51^

**Figure 1:**
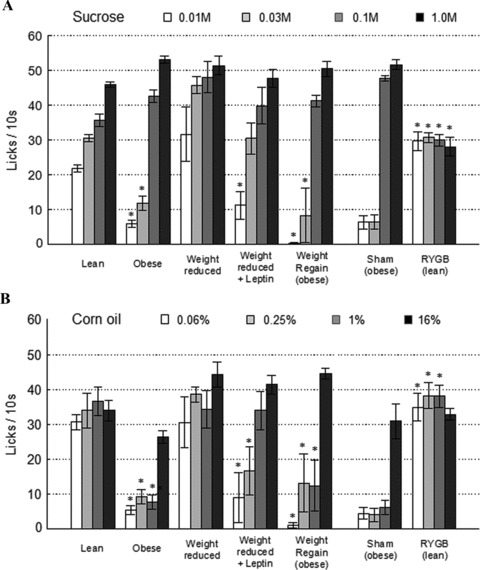
Brief access lick performance as a test of taste-guided liking of sucrose (A) and corn oil (B) in lean and obese rats. Lick performance was first compared between separate groups of chow-fed lean rats (*n* = 7) and high fat–fed obese rats (*n* = 7). Obese rats fed a high-fat diet throughout (*n* = 6) were then subjected to a cycle of weight loss (∼20% in three weeks by means of calorie restriction) and regain (two weeks, as shown in Fig. 3). During the weight-reduced state they received either saline or leptin (1 mg/kg, ip, 1 h before test). In another experiment, lick performance was assessed three to five months after sham surgery (*n* = 6) or RYGB surgery (*n* = 5). Note that the significantly reduced response performance to low concentrations of both sucrose and corn oil in the obese versus lean, weight-reduced + leptin versus weight-reduced, and weight-regain versus weight-reduced groups (*P < 0.05 compared with the same concentration). Also note the significantly increased response to the two low concentrations of sucrose and the three low concentrations of corn oil, but the significantly reduced response to the highest concentration of sucrose in RYGB rats compared to sham-operated rats (*P < 0.05, compared with the same concentration). Statistics are based on two separate ANOVAs for each taste stimulant, one for the sham-operated and RYGB rats and one for all other conditions, and Bonferroni-corrected multiple comparisons.

**Figure 2:**
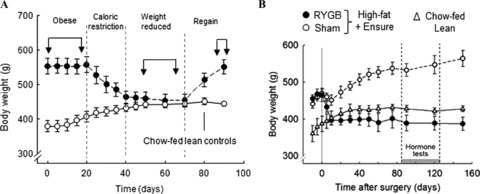
Body weight of rats used for testing reward behaviors. (A) Outbred Sprague–Dawley rats were either fed chow throughout (lean controls, *n* = 6) or were made obese by 12–16 weeks of high-fat feeding. One cohort of obese rats was then calorie restricted on a high-fat diet for three weeks and kept at a 20% lower body weight for four weeks, before the full amount of high-fat diet was restored and most of the lost body weight was regained. Arrows indicate the time of behavioral testing. (B) Outbred Sprague–Dawley rats made obese with a choice diet consisting of high-fat chow, Ensure, and chow were subjected to RYGB surgery (*n* = 6) or sham surgery (*n* = 8), and compared with age-matched, chow-fed lean controls. Behavioral testing was performed three to five months after surgery. Body composition was monitored throughout each experiment to verify the designations “lean” and “obese.”

We have started to more systematically evaluate the specific contributions of the obese state on measures of liking by comparing high-fat diet–induced obese outbred Sprague–Dawley rats with (1) never obese, lean rats, (2) formerly obese rats after calorie restriction–induced weight loss, (3) formerly obese rats after RYGB surgery–induced weight loss, (4) weight-reduced rats treated with leptin, and (5) formerly obese, weight-reduced rats after renewed weight gain.^52^,^53^ We also compared liking in the fed and fasted state of obese and lean rats.^52^ Furthermore, we have measured liking in genetically selected lines of obesity-resistant and obesity-prone rats before and after a period of high-fat feeding.^52^ As shown in Figure 1, while lean outbred Sprague–Dawley rats exhibit a near linear concentration-response curve in their brief-access sucrose- and corn oil–licking behavior, their obese counterparts show a right shift with less responding to the lowest concentrations but more responding to the highest concentrations. To determine whether the right shift was due to preexisting differences in the reward system or secondary effects of the obese state, the obese rats were then subjected to weight loss induced by restricting their access to a high-fat diet to 50–70%. After weight loss of about 20% over a period of three weeks, body weight was maintained at this lower plateau, and liking of sucrose and corn oil was reassessed in the fed state (Fig. 2). Weight loss resulted in a prompt shift of the concentration–response curve back to the left, not much different from the never obese, lean rats, suggesting that most of the difference in brief access responding between lean and obese SD rats was due to secondary effects of the obese state, not to preexisting differences in reward processing. The reversibility of the phenotype was further underscored by a renewed right-shift when the restricted animals were again allowed unlimited access to a high-fat diet (Fig. 1). Although we cannot rule out a weight loss-independent effect of the calorie-restriction procedure, it is impossible to separate such an effect from weight loss.

**Figure 3:**
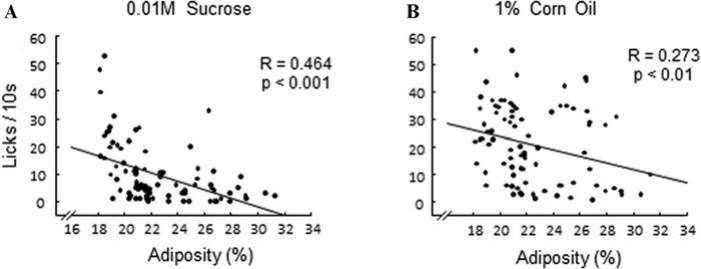
Regression analysis showing relationship between brief access lick performance and adiposity (as measured by NMR) across rats of all lean and obese conditions as shown in Figure 1. Note that lick performance for 0.01M sucrose (A) and 1% corn oil (B) was negatively correlated with adiposity.

Because human patients undergoing bariatric surgery were reported to decrease preference for fatty and sweet foods,^54^–^59^ we tested rats with sucrose and corn oil after RYGB or sham surgery in the brief access paradigm. While sham-operated rats that remained obese exhibited a similar right shift in the concentration–response curves for both sucrose and corn oil as observed in nonoperated obese rats, RYGB rats that lost about 20% of body weight five months after surgery (Fig. 2) exhibited a flat concentration–response curve, with much more responding to the lowest concentrations, but less responding to the highest concentrations compared with sham-operated rats for both tastants (Fig. 1). This response pattern was different not just from the sham-operated rats, but also from the never obese, lean, as well as the weight-reduced, formerly obese rats, suggesting that, in addition to effects of weight loss, the surgery had weight loss–independent effects. During the early postsurgical phase, before much weight loss has occurred, aversive conditioning could play an important role in reduced food intake in both humans^60^ and rats.^61^ At later postsurgical times, changes in the pattern of circulating gut hormones acting on the brain are thought to be major candidates for reduced appetite and food intake.^62^ However, as indicated by significant correlations between brief access responding to 0.01M sucrose as well as 1% corn oil and body fat content (as measured by NMR) across all lean and obese conditions of our studies, adiposity seems to be at least one factor determining the hedonic response to sweet and oily foods (Fig. 3).

In another model of obesity, the OLETF rat, which has a deficient CCK1-receptor, RYGB surgery also leads to selective reduction of brief access responding to high concentrations of sucrose,^63^ and a similar effect was shown in chow-fed rats after RYGB surgery.^64^

**Figure 4:**
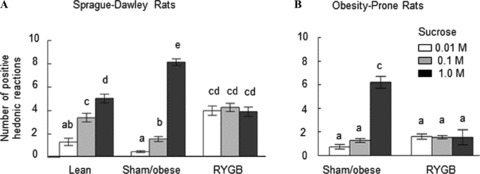
Number of positive hedonic orofacial reactions (Grill & Norgren's taste reactivity test) as a measure of liking of sucrose in Sprague–Dawley rats five months after RYGB or sham surgery and in age-matched, chow-fed lean rats (A), and in genetic lines of obesity-prone rats five months after RYGB or sham surgery (B). Bars that do not share the same letters are significantly (*P* < 0.05) different from each other (based on Bonferroni-corrected multiple comparisons following separate ANOVAs).

To confirm the changes in taste-guided licking behavior as measured in the brief access test with a more specific measure of hedonic liking, we compared chow-fed lean, RYGB, and sham-operated rats in the taste reactivity test that quantitates the positive hedonic orofacial reactions to the taste of sucrose. The results were almost identical to the brief access test, with sham-operated obese rats showing a right shift of the concentration–response curve and RYGB rats an essentially flat curve, with more responding to the lowest, and less responding to the highest, sucrose concentration (Fig. 4). These findings suggest that the brief access test measures something very similar to the taste reactivity test, and that this liking is reversibly changed by the obese state and additionally by some unknown mechanism induced by RYGB surgery, independent of weight loss.

**Figure 5:**
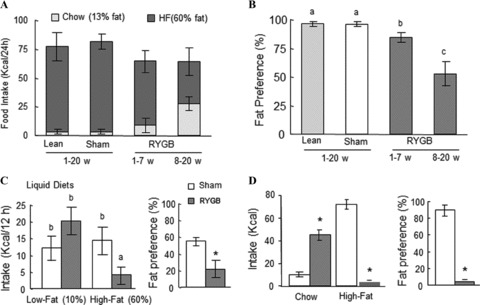
Gastric bypass surgery reduces fat preference in rats. (A, B) Gradual development of reduced fat preference after RYGB surgery in Sprague–Dawley rats as assessed with a choice of two complete solid diets low (13%) or high (60%) in fat. (C) Reduced fat preference measured three months after RYGB or sham surgery in Sprague–Dawley rats given 12-h access to two complete liquid diets, one low (10%) and one high (60%). Bars that do not share the same letter are significantly (*P* < 0.05) different from each other (based on Bonferroni-corrected multiple comparisons after appropriate ANOVA). (D) Almost complete avoidance of solid high-fat (60%) diet in chow-fed, nonobese Sprague–Dawley rats, three months after RYGB. *P < 0.05 compared to sham-operated rats.

Commensurate with the reported shift in preference away from fatty and sweet foods in human patients after bariatric surgery,^56^,^57^,^61^ we have also observed a shift in long-term preference for a low-fat over a high-fat diet after RYGB surgery in rats.^65^ There is a significant decrease in fat preference after RYGB compared to sham-operated rats when given a choice between a low-fat (10%) and a high-fat (30%) liquid diet in 12-h tests. There was also a gradual shift in preference away from the 60% high-fat solid diet to regular (low-fat) chow. This relative avoidance of fat was accentuated in lean rats fed regular chow before they were subjected to RYGB in that, while sham-operated lean rats readily switched to a high-fat diet, RYGB rats consumed very little of the high-fat diet (Fig. 5). These results are consistent with the idea that changes in liking, as measured in our acute test paradigms, translate into long-term preference for more healthy foods low in fat and sugar after RYGB surgery.

### Wanting

Berridge and Robinson's liking–wanting distinction^22^ has recently been considered for studying humans.^66^,^67^ In lean subjects, implicit wanting, as estimated by measuring reaction time in a forced choice paradigm, was not downregulated by food consumption in the same way as hunger, suggesting that it operates independent of homeostatic regulation.^68^ Increased implicit wanting of specifically sweet high-fat foods is found in subjects with high binge eating scores,^69^ suggesting that implicit wanting is a strong predictor of food intake. Using a different approach for estimating implicit wanting, Lemmens *et al*. found increased wanting for, and intake of, desserts and snacks in subjects with mild visceral obesity (BMI = 28 ± 1 kg/m^2^) compared with lean subjects.^70^

While these limited observations appear to agree with the commonly held view that obese subjects want palatable foods more than lean subjects, these cross-sectional studies cannot distinguish cause and effect. They cannot distinguish whether preexisting differences cause the obese phenotype or whether the obese phenotype alters reward functions and behavior. Furthermore, if food reward processing is particularly important for the consumption of highly palatable foods, to the extent that these foods can become addictive to some individuals,^71^,^72^ then it is also not clear whether, in analogy to drug addiction, repeated exposure to, and abstinence from, these addictive foods independently lead to alterations in reward processing.^73^,^74^ To determine the relative contribution of these three factors, they have to be selectively manipulated, which is not an easy task, particularly in human studies. The strongest evidence for predisposing, obesity-inducing differences in reward processing so far in humans has come from subjects carrying point mutations in genes known to be involved in brain reward processing. Comparing lean and obese subjects carrying different alleles of either the dopamine D2-receptor or mu-opioid receptor genes does reveal differences in behavioral and neural responses to palatable food.^75^–^78^ In a prospective study, the dorsal striatal response to food and subsequent six-month weight gain was measured in subjects either carrying or not the Taq A1 allele for the dopamine D2 receptor gene that leads to reduced receptor expression and likely diminished dopamine D2 signaling. While in carriers, weight gain was positively correlated with the magnitude of the food-induced striatal response, a negative correlation was found in noncarriers, suggesting that blunted activation of food reward circuitry to food and food cues increases the risk for weight gain if coupled with genetic risk for attenuated dopamine signaling, and exaggerated activation increases risk for weight gain in individuals without such a risk.^79^

In selectively bred lines of obesity-prone (OP) and obesity-resistant (OR) rats,^80^ as well as in Long-Evans rats,^81^ several differences in mesolimbic dopamine signaling and in progressive ratio responding, a behavioral measure of wanting, have been reported. We have used both progressive ratio lever press responding and the incentive runway paradigms to assess wanting in various rat models of obesity and weight loss. Using the incentive runway, it was shown that mice made hyperdopaminergic by genetically attenuating the dopamine transporter (DAT) function found the goal box significantly faster than wild-type mice.^82^ Thus, completion speed in the incentive runway is a measure of wanting and reinforcement learning to obtain a food or drug reward.^82^,^83^

**Figure 6:**
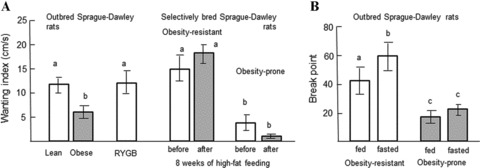
Motivation to obtain food reward (wanting) as measured in the incentive runway and progressive ratio lever press tests. (A) A wanting index was calculated as the mean completion speed averaged over trials 4–7. Note that in outbred Sprague–Dawley rats, high fat–fed obese rats showed reduced wanting compared to chow-fed lean rats and that wanting was restored after weight loss induced by RYGB surgery. Also, genetic lines of young obesity-prone rats exhibited reduced wanting compared to obesity-resistant rats. Eight weeks of high-fat feeding in these genetic lines did not produce further significant changes in wanting (B) Break point in the progressive ratio paradigm was significantly lower in obesity-prone versus obesity-resistant rats both in the fed and fasted condition. Bars that do not share the same letter are significantly (*P* < 0.05) different from each other (based on Bonferroni-corrected multiple comparisons after appropriate ANOVA).

Compared with lean chow-fed rats, high-fat diet–induced obese SD rats learned the task much slower, with initially significantly reduced completion speed, resulting in a lower *wanting index*^52^ (Fig. 6A). This was not due to nonspecific motor impairment, as the net running speed was not different, but obese rats spent significantly more time being distracted on the way, as indicated by significantly increased latency to leave the start box and duration of pauses and reversals. Similarly, young, chow-fed, genetically selected lines of OP rats exhibited a significantly reduced wanting index compared with their OR counterparts, which was further aggravated after eight weeks on a high-fat diet (Fig. 6A). Furthermore, progressive ratio lever press performance, as determined by the break point, was also lower in OP rats that had become overtly obese during 16 weeks on a high-fat diet compared to OR rats, both in the fed and fasted state (Fig. 6B).

Because it was reported that bariatric surgery patients have a diminished desire to eat^49^ and exhibit changes in striatal dopamine D2 receptor availability,^84^,^85^ we also measured wanting in our rat model of RYGB surgery. Sham-operated obese rats showed the familiar significant reduction of wanting in the incentive runway compared with lean controls (Fig. 6), and the slower completion time was due to increased duration of distractions, not to decreased running speed. Most importantly, this reduced wanting was fully reversed by RYGB surgery. RYGB rats wanted the food reward in the goal box just as much as lean control rats. Our findings are in agreement with the work of Davis and Benoit,^81^ demonstrating drastically reduced break points of progressive lever press responding for food reward in both high-fat diet–induced obese Long-Evans rats and young, genetically selected OP rats. This reduced wanting seen in obese rats by us and other authors seems at odds with the increased liking of the highest sucrose concentrations discussed above, as well as the increased implicit wanting of palatable foods observed in obese humans.^69^,^70^ Because the reward obtained in the incentive runway and progressive ratio tests was in the form of solid foods (Fruit Loops® and sucrose pellets, respectively), it could be that liquid sucrose, as used in the brief access and taste reactivity tests, is a more salient stimulus that may not have reduced wanting in obese rats. This possibility should be further explored in future studies. The apparent discrepancy in wanting of obese rats and humans could lie in methodological differences. Specifically, different levels of effort required to obtain the reward could be important. This is supported by the observation that genetically obese mice with MC4 receptor deficiency respond more when two lever presses were required (FR2) to obtain one small sucrose pellet, but they responded less when 50 lever presses were required (FR 50).^86^

**Figure 7:**
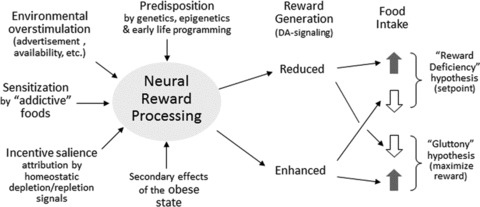
Schematic diagram showing the relationship between food reward and obesity.

Together, these findings suggest that the willingness of obese rats and mice to work for food depends on the effort required. Only if the effort is low, as for example in the brief access test or with low fixed ratio schedules of reinforcement, will obese rodents work for food reward. If the effort is higher, such as in the progressive ratio lever press and incentive runway paradigms, obese rodents stop working for food reward. Effort-dependent learning to work for food reward in operant schedules in normal weight rats has been comprehensively studied by Salamone and others, and it might have a neural basis in dopamine and adenosine signaling in the nucleus accumbens.^87^–^91^ That is, interference with dopamine signaling in this pathway makes normal weight rats work less hard for food rewards, the same effect that is seen in obese rats. This strongly supports the reward-deficiency hypothesis, which suggests that individuals with low dopamine signaling compensate by engaging in more eating, thereby restoring a set point for reward generation (Fig. 7). There is considerable evidence for decreased dopamine signaling in both in obese rodents^80^,^81^,^92^–^96^ and humans.^75^–^77^,^79^,^97^,^98^ However, because many studies simply compared lean with obese subjects, it has not been clear whether reward (dopamine) deficiency is the cause or consequence of obesity. One possible explanation of our findings of reversible reduced wanting in obese rats is that weight loss by either calorie restriction or gastric bypass surgery is able to restore dopamine signaling to normal levels, and experiments to test this hypothesis are underway.

Finally, the behavioral phenomenon known as *delayed discounting*—a measure of immediate over-delayed gratification—could play a role in the reduced wanting observed in our study, in that obese rats overconsume readily available high concentrations of sucrose and corn oil from the spout but under-perform if the reward is delayed in the runway or lever press paradigms. This possibility is supported by observations in obese women showing significantly greater delayed discounting,^99^ and children with difficulties in delaying gratification being more likely to become obese.^100^

## Conclusions

In conclusion, obesity is associated with complex alterations in food reward functions at the neural and behavioral level (Fig. 7). In general, obese subjects like and want palatable foods more than lean subjects, but these effects appear to be strongly dependent on the salience of the food stimuli and on the effort necessary to obtain these foods. Obese rodents *like* sucrose and corn oil more than their lean or weight-reduced counterparts when the concentrations are above a certain level—at low concentrations, they actually like sugar and fat less. Similarly, obese rodents *want* palatable food more only when it is easy to obtain, but they do not want to work for it. There is considerable evidence in both rodents and humans that reward deficiency with defective mesolimbic dopamine signaling is an important mechanism underlying at least some of these alterations in the obese. If reward generation through this system is diminished, either at an early age due to genetic and nongenetic predisposition or later in life due to secondary effects of diets high in sugar and fat and/or the ensuing obesity, increased food intake and the pursuit of other pleasures is used in an attempt to restore a reward set point. Thus, obesity-associated alterations of reward behaviors and neural functions are the result of both predisposing traits and secondary effects of repeated exposure to palatable diets and/or the obese state. The mechanisms by which repeated exposure and the obese state separately contribute to changes in reward behaviors, such as liking and wanting, and their respective neural pathways remain to be demonstrated.
